# Key indicators of obstetric and neonatal care in the Republic of Sakha (Yakutia)

**DOI:** 10.3402/ijch.v75.33956

**Published:** 2016-12-13

**Authors:** Tatyana E. Burtseva, Jon Øyvind Odland, Natalya I. Douglas, Antonina N. Grigoreva, Tatyana Y. Pavlova, Dgulustan A. Chichahov, Lena N. Afanasieva, Nurguyana S. Baisheva, Yana G. Rad, Mikhail I. Tomsky, Vitaly A. Postoev

**Affiliations:** 1 Yakut Scientific Center of Complex Medical Problems, Yakutsk, Russia; 2 Department of Community Medicine, Faculty of Health Sciences, UiT The Arctic University of, Tromsø, Norway; 3 North-Eastern Federal University, Yakutsk, Russia; 4 The Ministry of Health of the Republic of Sakha (Yakutia), Yakutsk, Russia

**Keywords:** pregnancy outcomes, birth rate, fertility, infant mortality, maternal mortality, circumpolar area, Russia

## Abstract

In the absence of a medical birth registry, the official statistics are the only sources of information about pregnancy outcomes in the Republic of Sakha (Yakutia) (RS). We analysed the official statistical data about birth rate, fertility, infant and maternal mortality in the RS in the period 2003–2014. Compared with all-Russian data, the RS had a higher birth rate, especially in rural districts. Maternal and infant mortality were also higher compared with all-Russian data, but had a decreasing trend. The majority of deaths occurred in the small level 1 units. We suggest that establishment of good predelivery transportation of pregnant women with high risk of complications from remote areas and centralization of risk deliveries with improved prenatal and neonatal care could improve the pregnancy outcome in Yakutia.

The climate conditions, specific daylight regimen, disparities in quality and accessibility of medical care are the important factors which could influence maternal and child health in circumpolar regions of the world. Key indicators, such as infant mortality, remain high in indigenous populations in many Arctic regions (1).

There is limited research about maternal and child health care in circumpolar regions of Russia. Only two regions have medical birth registries, namely Murmansk County and Arkhangelsk County (2). In the absence of registry-based studies, the official statistic is the only source of information. Thus, studies in the Komi Republic based on official data demonstrated a decline in infant and maternal mortality in 1997–2002 (3).

The Republic of Sakha (Yakutia) (RS) is situated in the north-eastern part of the Russia (Fig. 1). It occupies 3.1 million square km and 40% of the territory is above the Arctic Circle. The population was 956,000 in 2015, but the population density was only 0.3 people per square km (3). A large proportion of the rural population (35.8%) with predominantly indigenous inhabitants characterizes the RS (3). The settlements are remote and scarcely populated; moreover, they have weak communication and infrastructure. It is extremely difficult to organize medical care to avoid disparities especially among children and pregnant women.

**Fig. 1:**
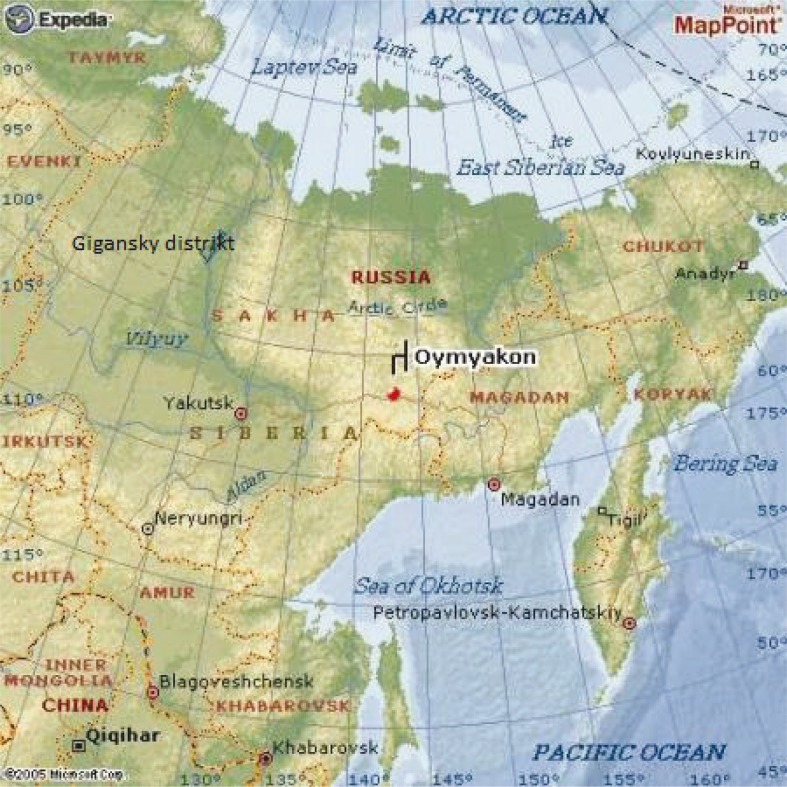
Map of the Republic of Sakha (Yakutia).

The aim of this study was to describe obstetric and neonatal care in the RS.

## Materials and methods

Data about birth rate, fertility, maternal and infant mortality in the RS in 2003–2014 were analysed using official statistical data reported by the Federal State Statistical Service (4–6). A comparison with all-Russian data was then performed.

## Results

### The system of obstetric care in the RS

The system of obstetric care in Yakutia is based on 30 maternity units in the central district hospitals (level 1), 6 urban maternity wards (level 2) and a perinatal centre, which is situated in Yakutsk, the capital of the RS (level 3). There are 379 hospital beds for pregnant women and mothers, 408 beds for pregnancy pathology and 503 beds for gynaecological patients. There are 5 obstetricians per 10,000 women and 31.2 obstetrics beds per 10,000 women of childbearing age.

### Birth rate and fertility

There were 17,074 newborns in the Republic in 2014 that corresponded with the birth rate of 17.8 per 1,000 (Table I). The birth rate increased in 2002–2014, higher than that of Russia as whole. Traditionally, birth rate was higher in rural areas. Thus, it was 24.3‰ in Zhigansky District (2014), but it was only 11.7‰ in the town of Neryungri (2014) (see Fig. 1).

**Table I:** Birth rate, fertility, maternal and infant mortality in the Republic of Sakha (Yakutia) and Russia in 2003–2014 (4–6)

	2003	2004	2005	2006	2007	2008	2009	2010	2011	2012	2013	2014
Birth rate, per 1,000 inhabitants												
Yakutia	15.0	15.5	14.3	14.4	16.1	16.2	16.8	16.8	17.1	17.5	17.5	17.8
Urban	14.5	15.0	13.8	14.1	15.1	16.0	16.7	16.5	15.6	16.6	15.5	15.3
Rural	15.8	16.3	15.0	14.8	17.5	16.0	16.7	17.4	19.9	19.9	21.1	22.4
Russia	15.0	15.5	10.2	10.4	11.3	12.1	12.4	12.5	12.6	13.3	13.2	13.3
Fertility per woman in child-bearing age												
Yakutia	1.87	1.91	1.73	1.72	1.91	1.90	1.97	2.00	2.06	2.17	2.17	2.25
Urban	1.66	1.71	1.58	1.61	1.72	1.82	1.88	1.86	1.77	1.89	1.78	–
Rural	2.35	2.36	2.01	1.97	2.31	2.08	2.18	2.30	2.68	2.81	3.15	–
Russia	–	–	–	–	–	–	–	–	1.58	1.69	1.71	1.75
Maternal mortality, per 100,000 livebirths												
Yakutia	56.2	47.6	22.1	36.6	26.2	19.5	12.5	24.8	12.2	29.4	18.0	29.4
Russia	31.9	23.4	25.4	23.6	22.0	20.7	22.0	16.5	16.2	11.5	11.3	10.8
Infant mortality, per 1,000 livebirths												
Yakutia	13.2	13.5	10.6	10.6	10.4	9.1	8.9	7.2	6.3	9.6	9.6	8.0
Urban	12.7	12.5	11.4	10.4	9.2	8.8	7.9	5.5	–	8.8	10.8	9.2
Rural	16.5	14.5	11.0	10.9	12.5	9.6	10.6	10.2	–	10.7	7.9	6.5
Russia	12.4	11.6	11.0	10.2	9.4	8.5	8.1	7.5	7.3	8.6	8.2	7.4

The total fertility rate was also higher than in all-Russian data. It correlated well with birth rate and was higher in the rural areas.

The majority of deliveries were in the level 2 units (56.5% in 2014), and it increased (from 51.6% in 2012). At the same time, deliveries at level 1 units decreased (from 34.0% in 2012 to 30.0% in 2014).

Besides the birth rate itself, the key issue is the proportion of normal deliveries (birth without any complications). According to the official statistics, the proportion of normal births increased from 28.8% in 2003 to 52.9% in 2014.

### Maternal mortality

The maternal mortality showed different trends in Russia compared with the RS: the all-Russian indicator had a threefold decrease in 2003–2014, while the republic indicator had great variation, with the highest in 2003 and the lowest in 2011 (see Table I). The difference between the Republic's and the all-Russian results was highest (threefold) in 2014. The maternal deaths mainly occurred in district hospitals (level 1). The main causes of maternal deaths were haemorrhagia, sepsis and eclampsia.

### Infant mortality

The infant mortality was 8.0 per 1,000 livebirths in 2014. It progressively decreased in the period 2003–2011 with a significant upward trend in 2012–2014 (see Table I). The neonatal mortality represented 60.3% of all infant deaths. The specific causes originating in the perinatal period were the main causes of neonatal death (76%); namely respiratory distress of newborns (including hyaline membrane disease) and neonatal pneumonia. These conditions should be considered as preventable causes of neonatal mortality, depending on the skills of doctors and midwives, to choose the right treatment and follow-up.

## Discussion

The trends in birth rate and fertility in the RS are similar to other countries within the circumpolar regions: the birth rate and total fertility were also higher in circumpolar territories in comparison with the whole country in USA, Canada, Denmark and Finland during 2000–2004 (7). A high birth rate, with high proportion of indigenous people, was observed in the rural areas. A total fertility rate higher than 2.0 is needed to keep the population stable. It is important to state that in rural areas of the RS, it was over 2.0 during the complete observation period, while in whole republic it achieved this number by 2010, and in the Russian Federation it is still less than 2.0.

The decreasing trend in infant mortality is a world-wide trend, also observed in circumpolar regions. However, the variation between the geographic areas is great: from 2.9 per 1,000 livebirth in Northern Finland to 12.7 per 1,000 in Greenland (7). The difference in perinatal mortality between national data and circumpolar areas varies significantly: from 8.0/1,000 between Denmark and Greenland to −0.4/1,000 between Finland and northern Finland. Interestingly, Finland was the country with the lowest perinatal mortality. However, a study from Quebec (Canada) showed that in spite of a decline in total infant mortality rate in country, the infant mortality among residents of indigenous communities did not decrease (8). Thus, it is important to monitoring this indicator separately in order to control and reveal the disparities in access to health care service among such groups. The difference in infant mortality between the all-country indicator and the republic one was not so high. It reached 1.2/1,000 in 2013 and decreased in 2014. The centralization of deliveries in the last years might improve this indicator in the coming years. Thus, perinatal mortality decreased from 10.9/1,000 in 2012 to 8.2/1,000 in 2014. Nevertheless, 41.3% of the preterm births in 2014 were at the level 1 units.

The maternal mortality rate decreased by two times in the RS and three times in Russia during the study period. However, a relatively small number of deaths might have huge impact on the statistical year-by-year variation in the RS. For example, three deaths resulted in 18.0 per 100,000 livebirths in 2013, and five deaths resulted in 29.4 per 100,000 livebirths. The majority of maternal deaths occurred in the small delivery departments of district hospitals, and they were associated with misjudgement of the situation. Early diagnosing of such conditions with subsequent transportation to the perinatal centre could prevent such cases.

## Conclusion

In comparison with all-Russian data, the RS had a higher birth rate as well as maternal and infant mortality. However, the majority of deaths occurred in small districts hospitals. The implementation of adequate pregnancy and delivery management with constant monitoring, including telemedicine, with subsequent preliminary transportation of pregnant women with high risk of complications from remote areas and centralization of such deliveries can improve the outcomes for both mothers and children.

The problems seem similar to those of other circumpolar regions. It is important to share experiences and develop joint international programmes and protocols for pregnant women and their outcomes in remote Arctic areas.
